# Dual Josephson Impedance Bridge: Towards a Universal Bridge for Impedance Metrology

**DOI:** 10.1088/1681-7575/ab948d

**Published:** 2020

**Authors:** Frédéric Overney, Nathan E. Flowers-Jacobs, Blaise Jeanneret, Alain Rufenacht, Anna E. Fox, Paul D. Dresselhaus, Samuel P. Benz

**Affiliations:** ∗ Federal Institute of Metrology METAS, Lindenweg 50, 3003 Bern-Wabern, Switzerland; † National Institute of Standards and Technology, Boulder, CO 80305, USA

**Keywords:** Impedance comparison, AC Josephson voltage standard, Josephson Arbitrary Waveform Synthesizer, AC coaxial bridge

## Abstract

This paper presents a full characterization of a Dual Josephson Impedance Bridge (DJIB) at frequencies up to 80 kHz by using the DJIB to compare the best available impedance standards that are (a) directly traceable to the quantum Hall effect, (b) used as part of international impedance comparisons, or (c) believed to have calculable frequency dependence. The heart of the system is a dual Josephson Arbitrary Waveform Synthesizer (JAWS) source that offers unprecedented flexibility in high-precision impedance measurements. The JAWS sources allow a single bridge to compare impedances with arbitrary ratios and phase angles in the complex plane. The uncertainty budget shows that both the traditional METAS bridges and the DJIB have comparable uncertainties in the kilohertz range. This shows that the advantages of the DJIB, including the flexibility which allows the comparison of arbitrary impedances, the wide frequency range, and the automated balancing procedure, are obtained without compromising the measurement uncertainties. These results demonstrate that this type of instrument can considerably simplify the realization and maintenance of the various impedance scales. In addition, the DJIB is a very sensitive tool for investigating the frequency-dependent systematic-errors that can occur in impedance construction and in the voltage provided by the JAWS source at frequencies greater than 10 kHz.

## I. Introduction

Impedance metrology makes intensive use of AC coaxial bridges for the realization of national metrology institutes’ capacitance, resistance and inductance scales at audio frequencies [1]–[3]. The type and complexity of the bridge depends on the type of the comparison. However, a shared property of these Wheatstone-like circuits is that, once the bridge is balanced, the measured impedance ratio is directly given by a voltage ratio [4]. The precise and accurate generation or measurement of this voltage ratio is therefore the cornerstone of impedance metrology.

State-of-the art impedance bridges [5]–[10] depend on ratio transformers or inductive voltage dividers (IVDs) to generate voltages whose ratio is accurate and stable. These bridges have been optimized over many years and are now able to compare impedance standards with an accuracy as small as 4 nΩ*/*Ω for specific impedance ratios and relative phase angles [11]. However, their main limitation is that the voltage ratio is fixed during fabrication when choosing the number of turns of the different transformer windings. Therefore, this high accuracy is possible only when impedance standards in a specific ratio (typically in 1:1 or 1:10 ratios) are compared.

Recently, high accuracy digital-to-analog converters (DACs) have been used instead of transformers or IVDs for the generation of the voltage ratio. These arbitrary voltage sources are used to compare impedance standards with arbitrary ratios and arbitrary phase angles [12]. However, the typical accuracy of these full digital bridges [13], [14] of about 2 *μ*Ω*/*Ω has not yet approached the accuracy that can be obtained with transformer-based bridges because of the limited accuracy and stability of the commercial DACs.

Programmable Josephson voltage standards (PJVS) can generate stable stepwise approximated ac waveforms, but such waveforms do not exhibit rms fundamental accuracy due to the presence of transients [15]. The first two-terminal-pair bridge based on PJVS synthesized voltages was demonstrated [16], [17] ten years ago. This bridge was used to compare impedances of the same type (R-R and C-C) with an accuracy comparable to transformer-based bridges over a frequency range from 20 Hz to 10 kHz. However, the large harmonic content of the PJVS waveform makes the comparison of impedances of different kinds (R-C, R-L or L-C) more challenging and limits the frequency range to a few kilohertz [4].

More recently, a new generation of full digital bridges have been developed that use two independent Josephson Arbitrary Waveform Synthesizer (JAWS) voltage sources to generate accurate arbitrary voltage ratios. A JAWS is a perfect digital-to-analog converter that produces a calculable, distortion-free voltage waveform with quantum-based accuracy over frequencies between a few hertz and 1 MHz or at DC. The first Dual Josephson Impedance Bridge (DJIB) clearly demonstrated that this type of bridge can compare any two impedances over a large frequency range (from 1 kHz to 20 kHz) with a demonstrated uncertainty below 0.5 *μ*Ω*/*Ω [18]. A separate realization of a two terminal version of a DJIB directly compared a 10 nF capacitance standard to the quantum Hall resistance with a combined relative uncertainty of about 0.01 *μ*Ω*/*Ω at a frequency of 1233 Hz [19].

In this paper, we present and evaluate an improved version of the first DJIB [18]. This bridge can be used at frequencies up to 80 kHz to compare any two impedances that are realized as four-terminal-pair standards [20]. After a brief description of the bridge in section II, we use the DJIB to evaluate in detail the performance of the JAWS system in section III, particularly focusing on accounting for bias-induced voltage errors. The bridge uncertainty budget is summarized in section IV.

The main results of this paper, an extensive series of validation measurements, are presented in section V. We perform two different types of measurements. First, we use the DJIB to measure different ratios (1:1, 1:10 and 1:1.29) of different types of known resistance standards with calculable frequency dependence at frequencies between 1 kHz and 80 kHz. These measurements include standards whose DC impedance is directly traceable to the quantum Hall impedance. The measured impedances agree with the expected values to within the Type A uncertainty of 0.02 *μ*Ω*/*Ω below 20 kHz and within the extended combined uncertainty of 0.2 *μ*Ω*/*Ω up to 50 kHz.

Second, we perform R-C and R-L bridge measurements using the DJIB and compare the resulting calibrations of the capacitance and inductance standards to the values obtained using the classical calibration chains. The results of the DJIB capacitance measurements are in good agreement with the classical chain to within the uncertainty of the DJIB u(*k*=1)=57 nF/F, which is slightly smaller than the uncertainty of the classical calibration chain in operation at METAS. The inductor measurement uncertainty u(*k*=1)=2 *μ*H*/*H are limited by temperature stability of the inductors, but clearly demonstrate the ability of the DJIB to compare any two types of impedances.

Finally, after the conclusion, an interested reader will find appendixes containing a comprehensive evaluation of the different uncertainty components along with an uncertainty budget covering the entire DJIB frequency range (Appendix A) and a more detailed description of the bridge (Appendix B).

## II. Bridge Description

The DJIB was designed to accurately determine the ratio of any two impedance standards defined as four-terminal-pair standards. The frequency ranges from less than 1 kHz up to 80 kHz and the maximum rms amplitude used in this work is 0.3 V. The bridge is fully computer controlled, though the operator must still manually change the connections between the impedances and the bridge. The DJIB can be divided into two distinct parts:

- The dual JAWS system, which generates two independent sine waves at the required frequency, amplitudes, and phases. The JAWS system is controlled by its own computer.
- The bridge, which is composed of detection and injection transformers as well as the different analog-to-digital convertors (ADCs) and DACs needed to measure the state of the bridge and trim the bridge balance, respectively. A second computer controls the bridge measurement sequence and iteratively tunes the JAWS output voltages and DACs to balance the bridge.

Although the DJIB is similar to other four-terminal-pair bridges [10], [18], a full description of the schematic, components and balancing procedure is given in Appendix B. In addition, the state equation of the bridge out-of-balance is developed in Appendix B. The state equation is used to determine the sensitivity of the bridge to the different balances.

### A. Schematics

**Figure 1.**
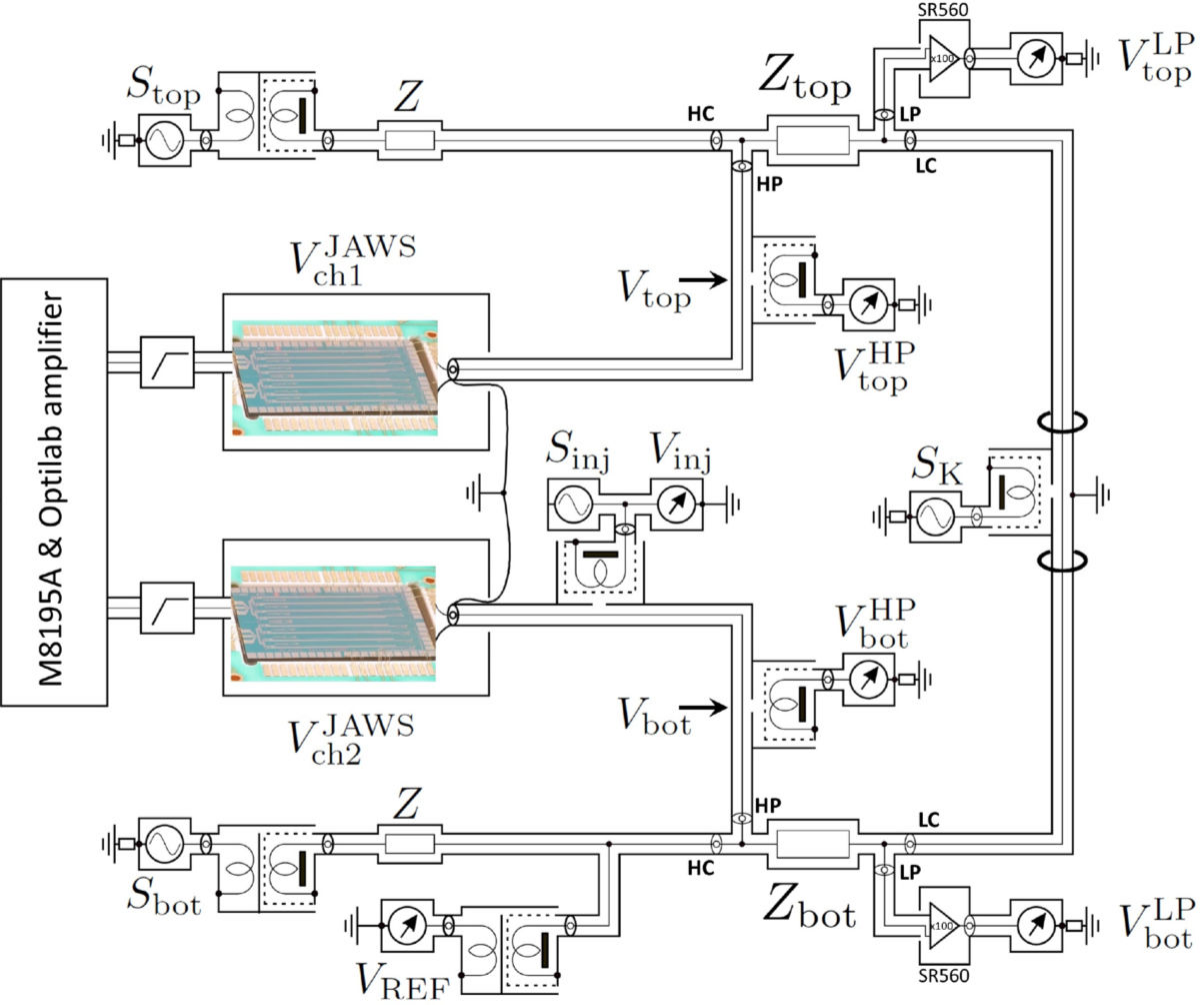
Schematic of the Dual Josephson Impedance Bridge (DJIB). The two four terminal-pair impedance standards to be compared (*Z*_top_ and *Z*_bot_) are connected in series. Once the bridge is balanced, the impedance ratio is equal to the voltage ratio.

Fig. 1 shows a detailed schematic of the DJIB. The two impedance standards to be compared are *Z*_top_ and *Z*_bot_. The four-terminal-pair definition of the standards is realized when the four detector voltages $V_{\text{top}\;}^{\text{HP}}$, $V_{\text{bot}}^{\text{HP}}$, $V_{\text{top}}^{\text{LP}}$ and $V_{\text{bot}\;}^{\text{LP}\;}$ are simultaneously zeroed. This is accomplished by iteratively adjusting first one of the JAWS sources $V_{\text{ch2}\;}^{\text{JAWS}\;}$ and then the DAC voltage sources *S*_top_, *S*_bot_, *S*_K_, and *S*_inj_ to simultaneously zero the detector voltages while minimizing *S*_inj_. Once this balance is established, the impedance ratio is directly given by the voltage ratio *V*_ratio_,

(1) $$\frac{Z_{\text{bot}\;}}{Z_{\text{top}\;}}=-\frac{V_{\text{bot}\;}}{V_{\text{top}\;}}=-V_{\text{ratio}},$$

where $V_{\text{top}}\propto V_{\text{ch}1}^{\text{JAWS}}$ and $V_{\text{bot}\;}\propto V_{\text{ch2}\;}^{\text{JAWS}\;}$ are the voltages defined at the center of the 1:100 detection transformers on the high potential (HP) arms of the bridge. Two supplementary digitizers are used to measure the voltage *V*_inj_ effectively applied to the primary winding of the 1:100 injection transformer and the reference voltage *V*_REF_ used to normalize the other measured signals (see Appendix B).

The DAC voltage sources and the detectors are the analogue outputs (AO) and the analogue inputs (AI), respectively, of commercially available high-performance, high-accuracy analogue I/O PXI card: the NI PXI-4461 and NI PXI-4462^1^. Each AO and AI channel has its own 24-bit converter, amplifier/attenuator and anti-aliasing filter. The maximum DAC/ADC sampling rate is 204.8 kSa/s.

In previous measurements [10], an error in the bridge balance condition was caused by cross-talk between the AO and AI channels of the NI PXI-4461 board. Therefore, the four critical AI channels that measure the balance condition are now provided by a NI PXI-4462 card without any AO channels. Two NI PXI-4461 cards are used to provide the AO channels and the less critical AI channels that measure the *V*_inj_ and *V*_REF_ voltages.

All three PXI cards are mounted in a PXI-1044 chassis. A shared 10 MHz clock is required to perfectly synchronize the AO/AI sampling rates to each other and the dual JAWS system clock so that the frequency of the auxiliary voltages and detectors used to balance the bridge match the frequency of the output voltages of the dual JAWS [21]. This clock is distributed to the PXI cards using the chassis 10 MHz back plane.

The impedance standards and bridge are further protected from the PXI-based circuits and external noise using isolation transformers, battery powered amplifiers, coaxial chokes, and auxiliary impedances *Z*. Leakage currents are eliminated using galvanic separation by adding double screened isolation transformers (either 1:1 or 1:100) between each AO channel and the impedance standards. Most of the AI channels are similarly isolated using transformers, except for the AI channels on the LP ports of the standards, which are isolated by placing low-noise, battery powered amplifiers (Model SR560, gain of 100) before the AI input. A single coaxial choke [22] is used in each mesh of the circuit to ensure current equalization and therefore reduce the effect of external interference on the bridge [2]. This specific choke configuration minimizes the current inequality and makes the bridge symmetric. Finally, auxiliary impedances *Z* are added to the high current arms (HC) to reduce the effect of the noise and drift of the *S*_top_ and *S*_bot_ AO channels on the balance condition [10].

### B. Dual JAWS source

The DJIB bridge is based upon the two voltages $V_{\text{ch1}\;}^{\text{JAWS}\;}$ and $V_{\text{ch2}\;}^{\text{JAWS}\;}$ that are generated by a dual JAWS system. In order to reduce cross-talk between the two voltage sources, the system is composed of two JAWS chips in two different probes that are co-located in a single liquid helium dewar. Electrically, each probe is a metal shield that surrounds the bias lines, output voltage leads, and chip. Cryoperm is used around the chip for magnetic shielding.

**Figure 2.**
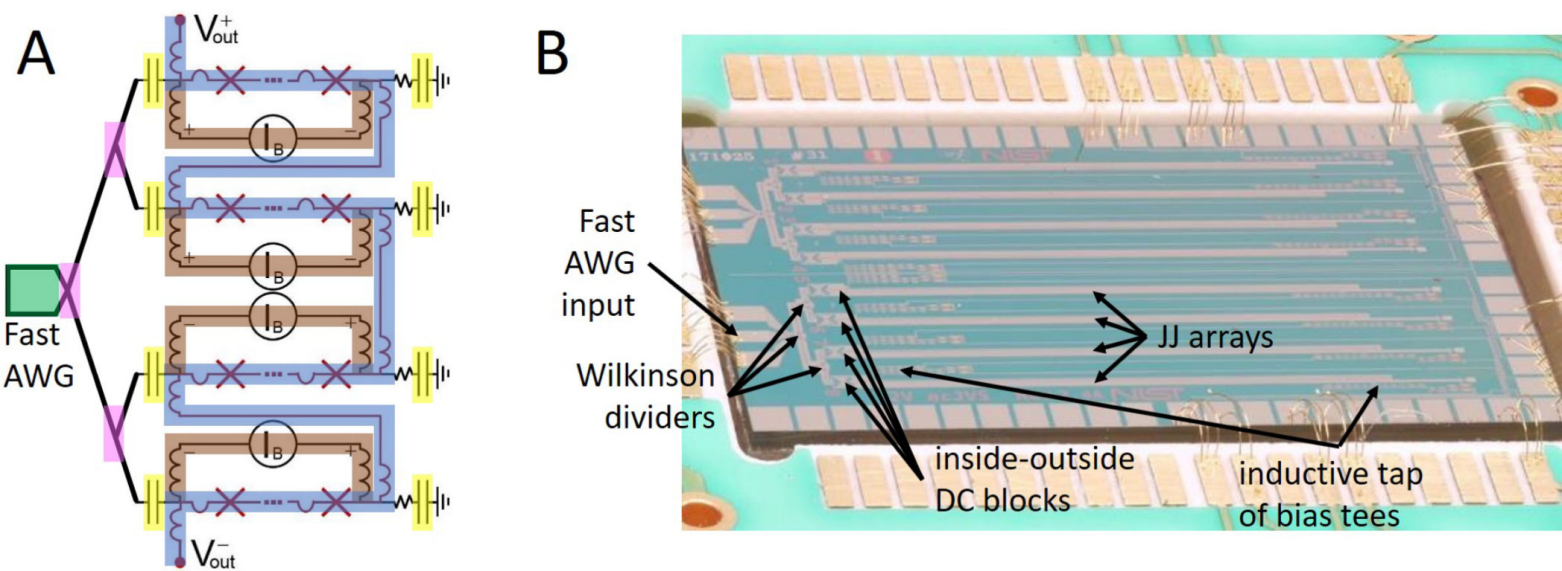
(A) JAWS source block diagram with fast arbitrary waveform generator (AWG) pulse bias (green), Wilkinson dividers (pink), inside-outside DC blocks (yellow), four JJ (red Xs) arrays connected in series using low-frequency bias tees (blue) and low-frequency isolated, floating current sources (brown). (B) Packaged JAWS chip which can be used to create two JAWS sources. The top and bottom halves of the chip are identical. The bottom half of the chip is labeled to correspond with the block diagram (A).

A single JAWS source is composed of a JAWS chip with many Josephson junctions (JJs), current bias electronics to apply fast, bipolar bias pulses to the JJs, as well as output voltage leads that transmit the voltage generated by the JJs (Fig. 2). When operating correctly, each input current bias pulse will cause each JJ to generate a single output voltage pulse. Due to the quantum properties of the superconducting state, the JJ voltage pulse has an integrated area *h/*(2*e*) that is dependent only on fundamental constants: the electron charge *e* and the Planck constant *h*. A DC voltage with calculable value *Nfh/*(2*e*) is generated from *N* JJs that are biased by current pulses with a constant repetition rate *f* [15].

To generate AC voltages with low-distortion and calculable frequency content, we use a delta-sigma algorithm to choose at each clock cycle whether to emit a positive bias pulse, negative bias pulse, or no pulse. In the measurements described in this paper, the pulse repetition rate of approximately 14.4 × 10^9^ pulses per second is much greater than the maximum waveform frequency of 80 kHz. This large oversampling ratio makes it relatively easy to generate patterns with a calculated spectral purity > 150 dBc below 1 MHz.

In detail, each source uses half of a JAWS chip and 51 240 JJs. Each JJ has a critical current of about *I*_*C*_ = 7 mA at 4.2 K and a normal junction resistance of *R*_*n*_ ≈ 0.4 mΩ. The JJs have amorphous niobium-silicide barriers with niobium superconducting leads and are arranged in vertical stacks of three JJs. The fabrication process and JJ properties are described in more detail in Ref. [23]–[25].

As shown in Fig. 2, the JJs (red crosses) are arranged in four series arrays that are embedded in the center conductors of four coplanar waveguides (CPWs). Each CPW is impedance-tapered from 50 Ω to a 21 Ω termination resistor. A single microwave pulse bias (green) is distributed to all four waveguides using two layers of Wilkinson dividers (pink) [26]. At low frequencies < 100 MHz, on-chip inside-outside DC blocks (yellow) are used to isolate the waveguides from each other and the pulse bias. The four JJ arrays can therefore be connected in series at low frequencies using on-chip bias tees (blue). The voltage output leads are the inner conductors of a pair of mini-coax that are connected to the two sides of the series connected JJ arrays. The outer grounds of the mini-coax are connected near the chip, and are grounded at that connection point using a separate ground wire that is connected to the DJIB ground at the top of the probe (a simplified version of this output voltage wiring is used in Fig. 1). A two-pole high-pass filter has also been placed between each pulse generator output and the probe head (see Fig. 1). This filter is composed of two pairs of inside dc blocks followed by 1 dB attenuators.

Each JJ array can also be connected to an isolated low-frequency current source (brown in Fig. 2). In this work, these current sources are used to test the stability of the JAWS sources before combining the JAWS with the bridge to create the DJIB. During DJIB operation the low-frequency current sources are disconnected at the top of the probe. These current sources are also often used to increase the RMS output voltages of the JAWS sources to 1 V each [26], [27]; in this mode of operation they are often called ‘compensation’ current sources. However, this mode of operation leads to a larger error in the output voltage due to the finite inductance of the JJ arrays [28]–[30]. In this work we therefore choose to operate in a ‘zero-compensation’ mode because this mode is simpler and leads to the lowest measurement uncertainties.

Operating in a ‘zero-compensation’ mode also requires that each of the high-frequency current pulses have a shape that has zero integrated area [31], [32]. For example, a typical ‘compensated’ current bias pulse has a gaussian shape with a width approximately equal to half the pulse repetition rate. On the other hand, ‘zero-compensation’ bias pulses are composed of a typical pulse surrounded on both sides by similar pulses with the opposite sign and half the magnitude. Despite the complicated shape, it is still possible to find a range of bias parameters where the JAWS source operates correctly and each input ‘zero-compensation’ bias pulse causes each JJ to generate one and only one output voltage pulse. It is worth emphasizing that the ‘zero-compensation’ bias input pulse has an integrated area approximately equal to zero while the output JJ voltage pulse has an integrated area exactly equal to *e/*(2*h*), that is, the JJ is a very nonlinear electrical element.

The goal of the small integrated area of the ‘zero-compensation’ pulse and additional high-pass filtering on the pulse bias line is to make the output voltage independent of the pulse amplitude by removing all of the bias signal power at the synthesis frequency. As discussed in the next section, this effort was not completely successful and we intend to improve the filtering in the future.

The main disadvantage of using the composite ‘zero-compensation’ pulse is that they are approximately triple the width of the typical pulse. This reduces the maximum JJ pulse rate by a factor of three and thus the maximum output voltage is also reduced by a factor of three. In this paper, the characterization and validation of the bridge was therefore performed at JAWS output voltages up to 0.3 V per source.

## III. Quantum Locking Range

The output voltage of a JAWS is determined by the timing of the voltage pulses generated by the JJs. When the system is operating correctly, every input bias pulse causes every JJ to generate a single output voltage pulse with the correct polarity. This locking of the output voltage pulses to the input pulses only occurs over a range of bias parameters that we call the quantum locking range (QLR) [33]. While there should be a QLR for all bias parameters, the DC current through the array and the input bias pulse amplitude are particularly relevant. In the literature, the DC current QLR is often referred to as the ‘flat spot’ or a ‘margins range.’

In this work, the DC current and pulse amplitude QLRs of each JAWS source are individually measured [34] at different amplitudes and frequencies. The optimum pulse shape and amplitude (at the center of the QLRs) for each channel are determined before each measurement campaign. During the campaign, these pulse parameters are the same for all output waveforms on a given channel, that is, independent of the waveform frequency, magnitude, or phase. During the initial setup phase, we measured the DC current offset QLR to be greater than 1.2 mA at an rms output voltage of 0.3 V and 1 kHz in the ‘zero-compensation’ mode. The DC current offset QLR did not depend strongly on frequency and increased as the rms output voltage was decreased, which is typical of ‘zero-compensation’ waveforms.

However, it is not sufficient to just measure the JAWS QLRs during the initial setup phase with the JAWS sources disconnected from the bridge. When the JAWS sources are integrated into the bridge, the presence of current noise induced either by undesired ground loops or by other voltage sources used in the bridge can reduce the QLR. In the worst-case scenario, these perturbations could jeopardize the quantum-based accuracy of the generated voltage ratio.

Therefore we also perform QLR measurements using the bridge as a detector to verify that the JAWS is operating correctly [33]. After balancing the bridge, we dither the bias parameters and measure the effect of the dither on the ratio. Since the JAWS DC bias wires are disconnected during bridge operation, we individually dither the pulse amplitude of each channel to test the most important accessible bias parameter.

**Figure 3.**
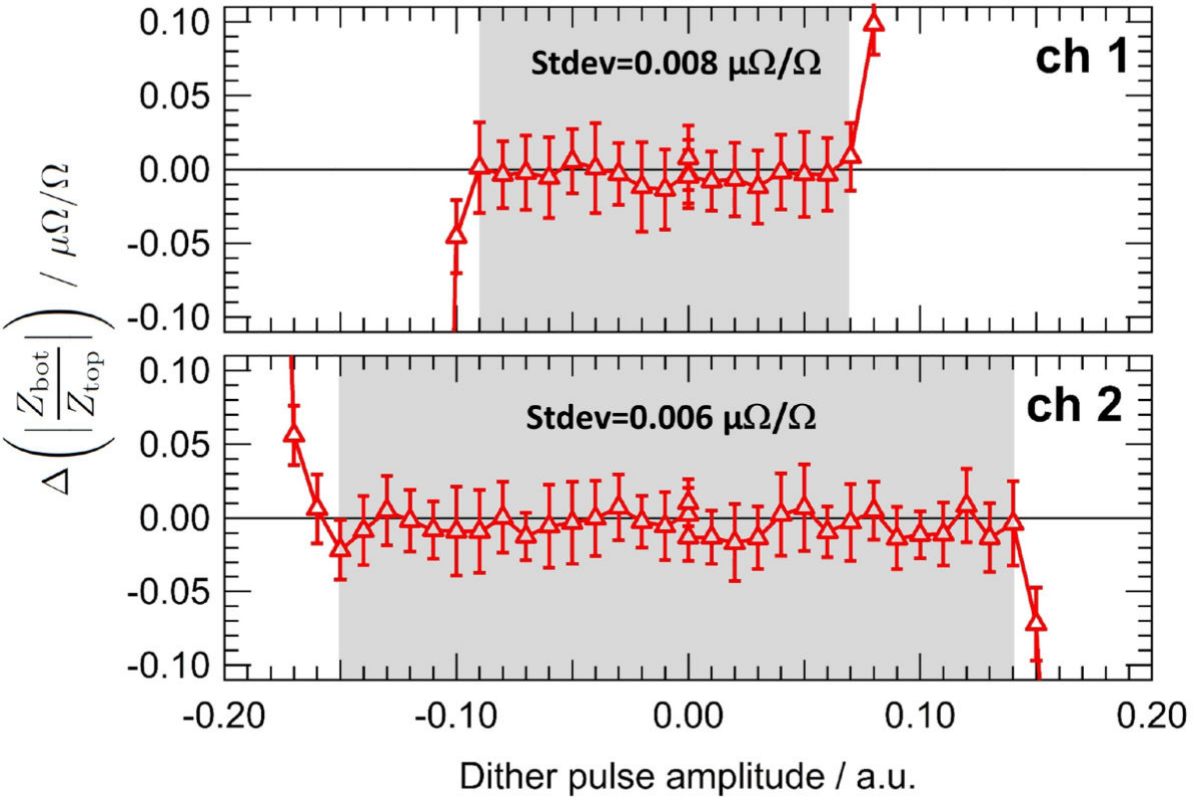
Variation of the modulus of the measured impedance ratio when the pulse amplitude of channel 1 (top) or channel 2 (bottom) deviates from the center of the plateau. The measurements are performed in the direct configuration, at 1 kHz and an rms voltage of 0.3 V. The uncertainty bars indicate the type A uncertainty of the bridge balance. The gray zone indicates the QLR. Within the gray zone, the measured impedance ratio is independent of the pulse amplitude and the standard deviation of the measured modulus of the impedance ratio is smaller than 0.01 *μ*Ω*/*Ω.

**Figure 4.**
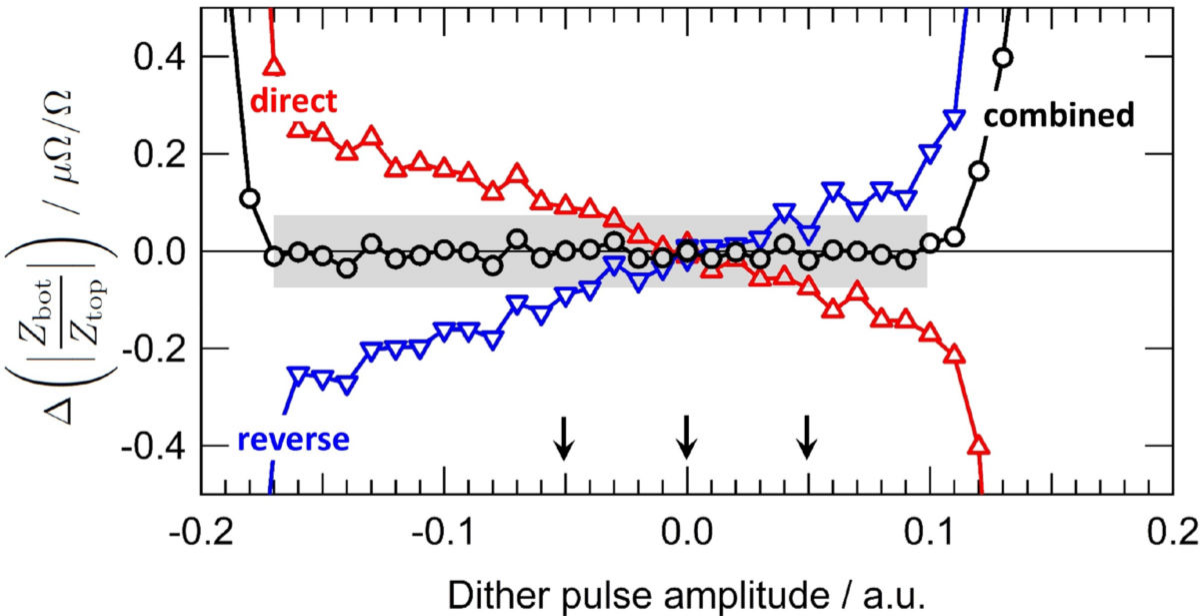
Measurement of the pulse amplitude QLR of channel 2 at 80 kHz and 0.3 V. Within the gray zone, the standard deviation of the modulus of the combined result is 0.014 *μ*Ω*/*Ω. The three arrows indicate the values of the dither amplitude used in the mini-QLR measurements (see text for details).

Fig. 3 shows the effect of changing the JAWS pulse amplitude on the modulus of the impedance ratio measured at 1 kHz and at a rms amplitude of 0.3 V. The impedance standards compared are two calculable resistors of 12.906 kΩ. The pulse amplitude parameter (in arbitrary unit) can be varied from 0 to 1 and the center of the QLR is 0.46 for the channel 1 and 0.7 for the channel 2. The gray zones indicate the effective pulse amplitude QLRs of the DJIB. Within these gray zones, the standard deviation of the modulus of the measured impedance ratio is smaller than 0.01 *μ*Ω*/*Ω. The independence of the measured impedance ratio and the pulse amplitude over the QLR confirms that the JAWS is working correctly as a quantum-based standard with a single output JJ pulse per input bias pulse and that voltage errors caused by the pulse bias are negligible. At higher frequencies we begin to observe errors caused by the pulse bias. In Fig. 4 we show similar QLR measurements to those discussed in the previous paragraph, only at a frequency of 80 kHz. In this case, we just show channel 2 (channel 1 behaves similarly), but we include the results from both the direct and reverse bridge configurations (red and blue, respectively). At this higher frequency, there is a slightly reduced QLR but more importantly both the modulus and the phase of the measured impedance ratio depends slightly on the pulse amplitude within the QLR. The linear dependence over the QLR implies that this error is caused by the power in the pulse bias at the synthesis frequency (as was discussed in the previous section), that is, at 80 kHz in this measurement, indirectly coupling to the JAWS output voltage leads. Also, the magnitude of the effect can be increased by removing high-pass filters between the pulse generator and the JJ array (see Fig 1). In the future, we intend to improve the filtering to reduce this error.

However, the impedance reversal procedure used in the DJIB means that the final, combined impedance ratio (black in Fig. 4) is less sensitive to JAWS voltage errors caused by the indirect coupling of the bias pulses into the output voltage leads. This insensitivity depends on three reasonable assumptions: (a) the bias signal at the synthesis frequency is linearly dependent on the JAWS output voltage, (b) the indirect coupling between the bias signal at the synthesis frequency and the voltage output leads is linear and constant, and (c) the voltage error is small (for a detailed calculation and explanation see Appendix B). The measured data in Fig. 4 are consistent with these assumptions. Within the gray zone, the standard deviation of the combined modulus is 0.014 *μ*Ω*/*Ω.

The full measurement sweeps over the entire QLR presented in Fig. 3 and 4 are too time consuming to be systematically performed during each ratio measurement. However, we do systematically perform ‘mini-QLR’ measurements [33] during every ratio measurement in both the ‘direct’ and ‘reverse’ configurations. During these regular mini-QLR measurements, the impedance ratio is measured six times per impedance configuration: each channel is treated separately, and a measurement is made at the optimal pulse amplitude in the center of the QLR as well as with a small positive and negative offset. During this dither, the other channel is kept at the optimal value. We choose a pulse amplitude dither offset value of 0.05 (arbitrary units) per channel (arrows in Fig. 4 for channel 2) so that we are dithering over a significant fraction of the QLR, but do not expect small environmental changes to cause problems. When the standard deviation of the six different measured combined impedance ratios is significantly larger than the type A uncertainty of each measurement, the JAWS system may not be operating properly and supplementary investigations are therefore required.

Although the slope *s*_ch2_ in Fig. 4 is measurable with a good signal-to-noise using the DJIB, the magnitude of the slope is small and it would be difficult (if not impossible) to observe such small variation of the JAWS’s output voltage using a thermal transfer standard [35], [36]. Moreover, the measurement of the slopes *s*_ch2_ or *s*_ch1_ (see Appendix B, section D) does not require knowing anything about the impedance standards; it only requires that they are stable during the ratio measurements. Therefore, the DJIB is also a useful characterization tool for the JAWS system.

**Figure 5.**
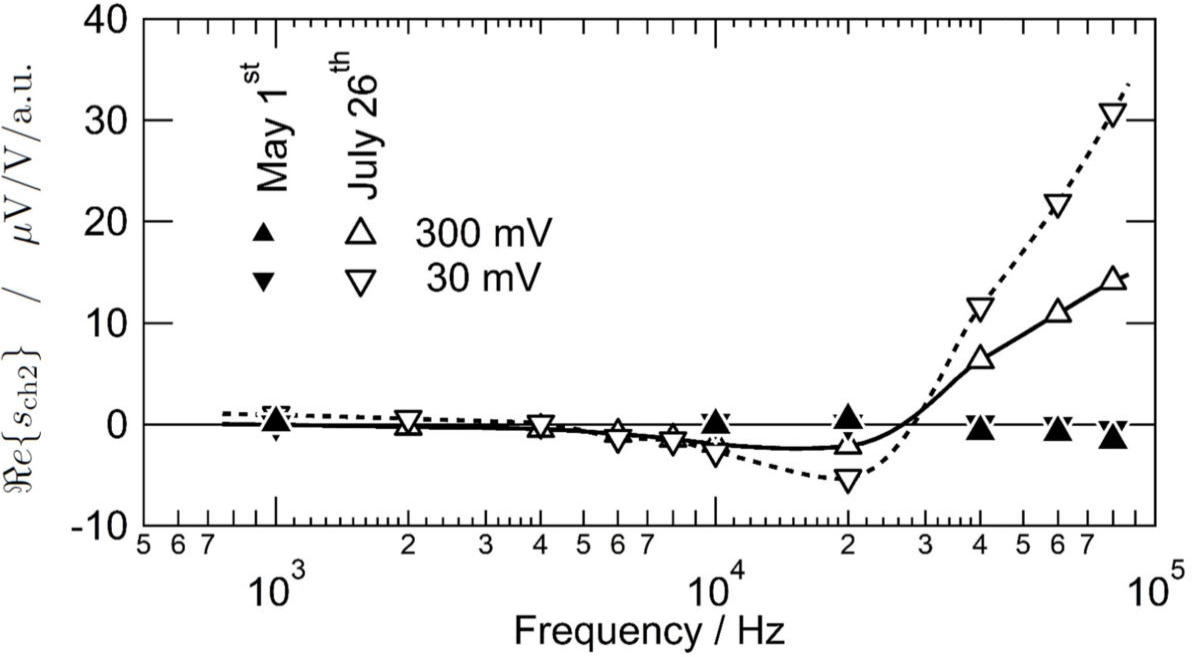
Frequency dependence of the real part of the slope of the channel 2 pulse amplitude QLR at two different output rms voltages, 300 mV (upward triangle) and 30 mV (downward triangle), and repeated at two different times: May 1^st^ (solid symbols) and July 26^th^ (open symbols) 2019. The solid and dashed lines are only guides for the eye.

Fig. 5 shows the real part of the slope *s*_ch2_ determined from the pulse amplitude QLR measurements performed at frequencies between 1 kHz and 80 kHz and at two different output rms voltages (300 mV and 30 mV). We observe a significant change in *s*_ch2_ between May and July 2019. The two measurements correspond to two different measurement campaigns; between the two campaigns, the DJIB system was moved to another laboratory and stored for a month with all the components at room temperature.

The measurements made during the first measurement campaign in May show a small slope *s*_ch2_ over the whole frequency range. Moreover, *s*_ch2_ is largely independent of the JAWS output voltage level, which is required for the effect to be removed by combining the ‘direct’ and ‘reverse’ measurements (see Appendix B-D). The data in Fig. 3 and 4 were also taken during this May measurement campaign. For the remainder of this section we will concentrate on channel 2. However, similar results were observed on channel 1.

During the second measurement campaign, we observed a slope *s*_ch2_ with a larger magnitude, more frequency dependence, and most significantly a dependence on the JAWS output voltage at frequencies > 10 kHz. While the real part of the slope is still small at low frequencies, the value becomes slowly more negative before suddenly becoming positive and increasing sharply with frequency above 20 kHz. A serious concern is that the slope becomes dependent on the JAWS output voltage at frequencies > 10 kHz. This implies that the assumptions about system linearity discussed earlier (and in Appendix B-D) are no longer accurate and the combined impedance ratio is no longer expected to be independent of pulse amplitude (see Eq. B.25) when comparing unequal impedance standards. In such a case, the uncertainty component related to the slope will increase significantly.

The origin of the change in the frequency and voltage dependence of the slope is not yet fully understood. However, the most likely source of the problem is a change in the properties of the pulse amplifier. The large pulse magnitudes required by the JJ arrays of > 26 dBm at the pulse amplifier output mean that the amplifier is usually operating near its 1 dB saturation point. A small increase in the required power or change in the amplifier characteristics could effectively increase the amplifier’s non-linearity, which would make the bias signal at the synthesis frequency much larger and non-linearly dependent on the JAWS output voltage. This does not explicitly explain the frequency dependence of the slope, but if the amplifier is operating with significant non-linearity, then a complicated frequency dependence is possible. This is particularly true if the pulse bias is large enough that it causes the amplifier power or biases to oscillate at the synthesis frequency. Also, most models for the coupling between the bias signal and the output leads would imply a larger coupling at higher frequencies.

## IV. Uncertainty Budget

The evaluation process of every uncertainty component of the DJIB has been performed in the frequency range from 1 kHz to 80 kHz. Many components of the uncertainty budget are similar to the one of the digitally assisted bridge (DAB) [10] and only their improvement is discussed here. Moreover, a detailed description of the evaluation of the main components is given in Appendix A.

**Figure 6.**
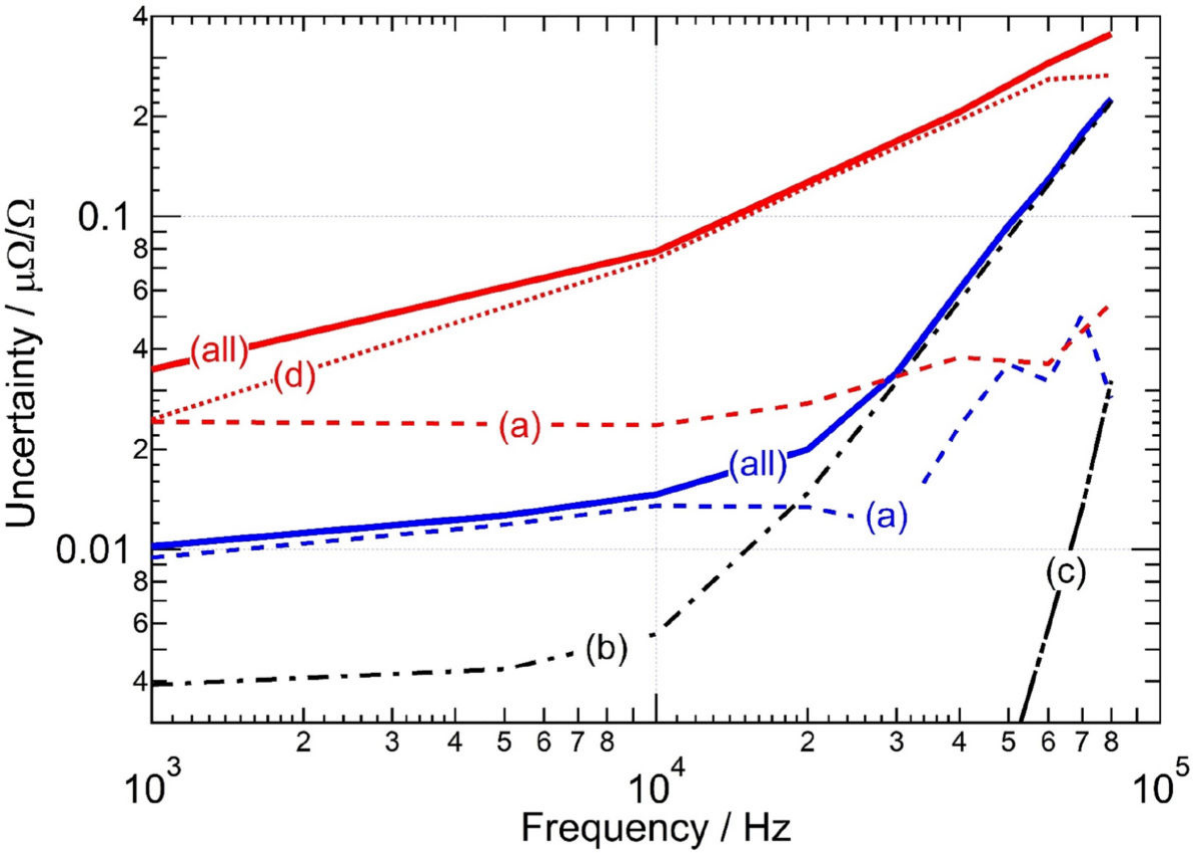
Relative standard uncertainty (*k*=1) components of the modulus of impedance ratio when comparing impedance in 1 to 1 (blue curves) and 1 to 10 (red curves) ratios. The black curves are common to both ratios. (a) Bridge resolution. (b) Cable corrections. (c) Injection chain. (d) QLR slopes. (all) combined standard uncertainty. The numerical values are given in Table I.

Fig. 6 shows the relative standard uncertainty (*k*=1) components of the modulus of impedance ratio when comparing impedance in 1 to 1 (blue curves) and 1 to 10 (red curves) ratios; some uncertainty components are independent of the impedance ratio (black curves). The numerical values for these uncertainty components are given in Table I for 1 kHz, 10 kHz and 80 kHz.

- Curve (a) represents the resolution of the bridge, which determines the smallest measurable variation of the impedance ratio. It is limited by the noise level of the different balances and by the sensitivity of the impedance ratio to those balances. It is the Type A uncertainty of the bridge.
- Curve (b) represents the uncertainty related to the cable corrections. There are two types of cable correction to take into account. The first is the loading effect of the cable between the voltage outputs on the JAWS chips and the detection transformers on the HP arms, where *V*_top_ and *V*_bot_ are defined. The second is the classical correction related to the cables needed to connect the impedance standards to the bridge [2], [20]. When the standards are exchanged at the level of the standards’ connectors, the connecting cables are effectively part of the bridge and the effects of both cable corrections are removed by combining the ‘direct’ and ‘reverse’ measurement results (see Eq. B.13). Nevertheless, an uncertainty has to be evaluated to account for possible variation of cable characteristics between the two phases of the measurement. This component is the same for both the 1:1 and 1:10 ratio, but becomes the dominant uncertainty component in the 1:1 comparison at frequencies above 20 kHz.
- Curve (c) represents the uncertainty related to the accuracy of the injection chain. This component has significantly been reduced in comparison to the DAB. Indeed, the ratio of the Josephson voltages can be adjusted to limit the amplitude of the injected component to less than 20 *μ*V/V at every frequency. Moreover, the unique ability of the DJIB to generate arbitrary voltage ratios makes the calibration of the whole injection chain straightforward.
- Curve (d) represents the uncertainty related to the QLR problems discussed in the previous section. This uncertainty component is negligible when the impedance ratio is close to 1:1, but is the dominant component when the impedance ratio is 1:10.

In earlier measurements [10], the uncertainty component related to the cross-talk between the DACs and the ADCs dominated the uncertainty budget of the DAB. However, the use of one NI PXI-4462 board (instead of two NI PXI-4461 boards) has reduced this uncertainty component to a negligible level. Other uncertainty components, including those related to the efficiency of the coaxial chokes and to the limited resolution of the 24-bit ADC, are also negligible and not included in Fig. 6 or Table I.

## V. Validation

An extensive set of impedance comparisons has been performed to validate the ability of the DJIB to perform impedance calibration over the full complex plane. For this purpose, the results obtained with the DJIB were compared to the results determined either from the calculability of the standards (for the R-R comparisons) or from the traceable reference values obtained using the classical calibration chains (for the R-C and R-L comparisons).

### A. R-R comparison

Table II lists the different resistance standards used for the resistor comparisons. They are calculable resistance standards for which the relative variation *δ*_ac*/*dc_ of the resistance and the time constant *τ* can be calculated from the geometrical dimension and material properties of the standard.

G1 and G2 are two commercially available quadrifilar resistance standards [37] of 12.906 kΩ. They are temperature regulated and their DC value is regularly calibrated against the DC quantum Hall resistance *R*_K_ = *h/e*^2^. Between two DC calibrations the resistance has a linear drift and the DC value can be predicted with an uncertainty smaller than 0.005 *μ*Ω*/*Ω. The calculated frequency dependence of the quadrifilar resistance standards has been validated through different inter-comparisons up to a frequency of about 5 kHz [5], [7].

The Haddad type resistors are home-made standards [38], [39]. H1a and H1b have a nominal value of 1.2906 kΩ, i.e., 1/10 the resistance of the quadrifilar standards, and H2a and H2b have a nominal value of 1 kΩ. The Haddad-type standards are not temperature regulated and their DC values are not as predictable as the quadrifilar standards. The calculated frequency dependence of the H2a standard has been validated through a bilateral comparison up to a frequency of 20 kHz [40].

**Figure 7.**
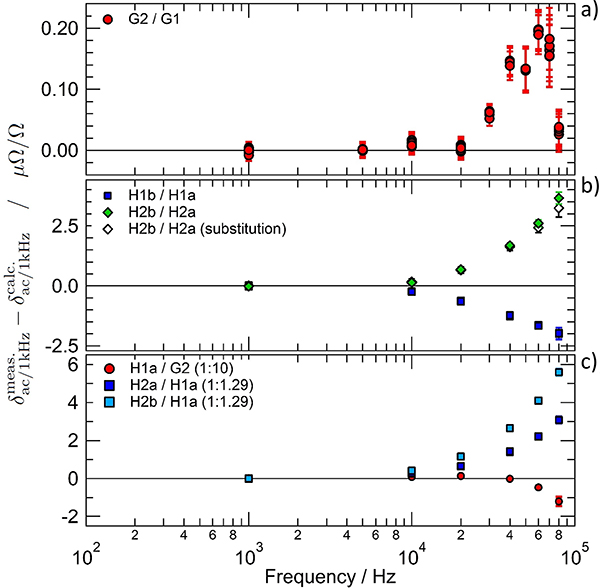
Difference between the measured and calculated frequency dependence of the modulus of the impedance ratio of calculable resistance standards. Calculable resistance standards with a nominal value of 12.906 kΩ, 1.2906 kΩ and 1 kΩ have been used. In the top plot (a) the uncertainty bars correspond to the Type A uncertainty only. In the other plots (b,c) the bars represent the combined standard uncertainty (*k* = 1). It is also worth noting the change in the vertical scale between the different plots.

These impedance standards can be combined to form impedance ratios of different values: 1:1, 1:10 and 1:1.29. The impedance ratios have been both measured and calculated at frequencies between 1 kHz and 80 kHz. Fig. 7 shows the difference between the measured and calculated variation of the modulus of impedance ratio from the value at 1 kHz *δ*_ac/1kHz_.

The results for the 1:1 ratio are shown in Fig. 7a,b and those for the 1:10 and 1:1.29 ratios are plotted in Fig. 7c. In Fig. 7a, the uncertainty bars represent only the type A standard uncertainty. In the two other plots the uncertainty bars correspond to the combined standard uncertainty with a coverage factor of *k* = 1.

A good agreement between the calculated and the measured impedance ratio G2/G1 is observed over the whole frequency range. Although deviations larger than the Type A uncertainty are observed above 20 kHz, the maximum value remains within the extended combined uncertainty, which is *U*(*k* = 2) = 0.2 *μ*Ω*/*Ω at 50 kHz. At each frequency, six measured values have been obtained during the mini-QLR procedure. The scatter of these values (plotted separately at each frequency in Fig. 7a) is smaller than the type A uncertainty, confirming that the JAWS sources are operating correctly with quantum-based accuracy.

When home-made Haddad resistors are involved in the comparison, a good agreement is only obtained for frequencies below 10 kHz. Above this frequency, the difference between the measured and the calculated values increases and becomes larger than the extended uncertainty. This deviation can be explained by either an unaccounted frequency dependent bias in the bridge or by a supplementary frequency dependent term that was overlooked in the model used for the theoretical calculation of the frequency dependence of the resistors [38].

An unaccounted frequency dependent bias of the DJIB can be ruled out by a substitution comparison, at least within the present uncertainty limits. Indeed, the combination of the two 1:1.29 comparisons (H2a/H1a and H2b/H1a) leads to the determination of the ratio H2b/H2a by substitution (Fig. 7b, open diamonds). The main advantage of a substitution comparison is that all the biases of the measuring system are cancelled out. The good agreement achieved between the determination of the ratio H2b/H2a obtained from a direct comparison and from substitution measurements proves the absence of a frequency dependent bias of the DJIB.

A flaw in the modelling of the Haddad resistor is a more plausible explanation, since the Haddad resistors were specifically designed to supply a low frequency calculable frequency dependence for the realization of the R-C chain. Therefore, design details that have a negligible effect at low frequency may have been neglected. However, this measurement does show that the high accuracy of the DJIB can be used to characterize these small effects that cannot be measured by any other higher frequency bridge [41]–[43].

In the future, comparisons of calculable resistance standards specially designed for high frequencies should be used to further characterize the DJIB performance [42], [44]. In the meantime, the calculated frequency dependence of the Haddad resistance standards will be used above 10 kHz with an extra uncertainty component taking into account the deviations observed in Fig 7.

### B. R-C comparison

In impedance metrology, the most challenging uncertainty requirements are found in the realization of the R-C chain. The measurements presented in this section therefore represent the most stringent test for the DJIB.

Dedicated quadrature bridges, designed specifically for this task, have been optimized over the last 50 years [45]–[52]. Nowadays, 100 pF capacitance standards can be calibrated in terms of *R*_K_ with a relative uncertainty of only a few parts in 10^8^ [8], [11]. Although a few versions of transformer-based quadrature bridges have been designed to be operated at multiple frequencies [9], [53], [54], most of them only work at a single frequency: 1233 Hz for comparison of 10 nF to 12.906 kΩ and 1592 Hz for comparison of 10 nF to 10 kΩ.

#### 1) Calibration of 100 pF at 1233 Hz:

**Figure 8.**
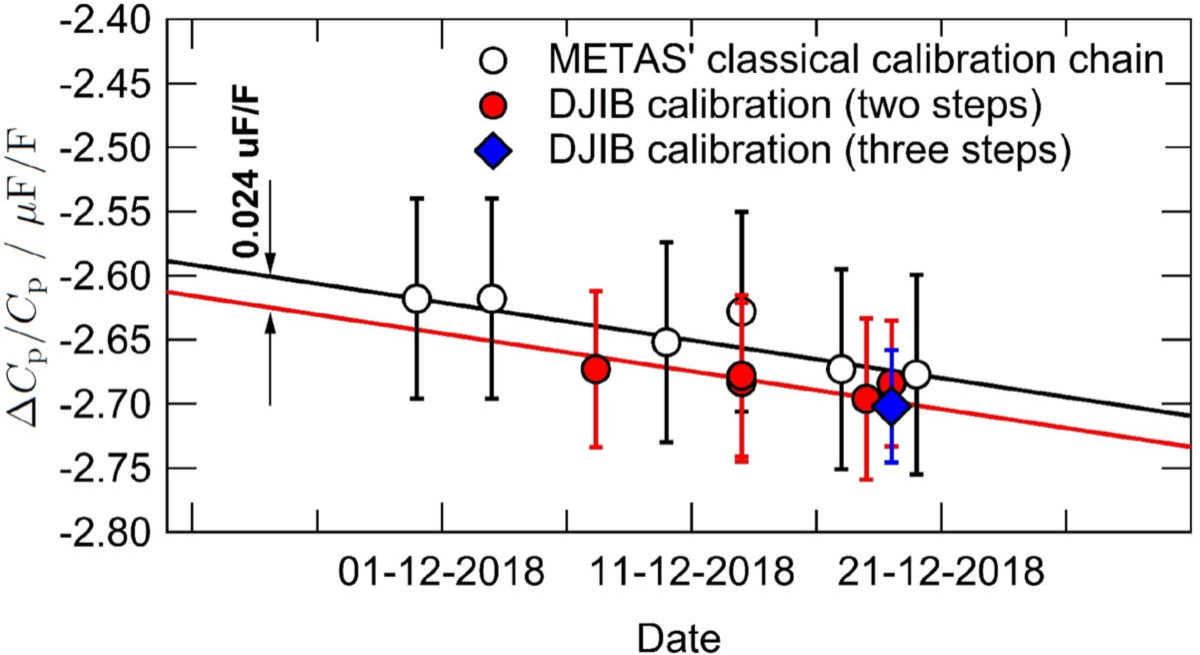
Calibration of a 100 pF capacitance standard in terms of *R*_K_ using the classical calibration chain and using the DJIB. The measurements were performed at a frequency of 1233.147 Hz. The uncertainty bars correspond to the combined standard uncertainty (*k* = 1).

Fig. 8 shows the results of the calibration of 100 pF capacitance standards performed at 1233.147 Hz over a 20 day period. Two calibration chains have been used: the METAS classical calibration chain using a quadrature bridge and a 1:10 ratio bridge (described in [8]), and the new calibration chain that only uses the DJIB.

The DJIB calibration chain can be made using either a three-step or a two-step comparison procedure. In the three-step procedure, a 10 nF standard is calibrated by comparison to the reference resistance standard G1 in a 1:1 R-C comparison. Then two 1:10 C-C comparisons are performed to calibrate the 1 nF and the 100 pF standards.

In the two-step procedure, the reference resistance standard G1 is directly compared to a 1 nF standard in a 1:10 R-C comparison. Only one 1:10 C-C comparison is then needed to obtain the value of the 100 pF standard.

The results obtained with both the three-step and the two-step procedures are in very good agreement, showing the high consistency and reproducibility of the DJIB measurements. Even more convincing is the good agreement between the results obtained with the well-established classical calibration chain and the DJIB. The small offset of 0.024 *μ*F/F observed between the two set of results is well within the combined uncertainties *u*(*k* = 1) = 0.06 *μ*F/F. Moreover, this offset partly corrects the 0.05 *μ*F/F bias of the METAS classical calibration chain revealed during the last comparison CCEM-K4.2017 [8].

Finally, we note that the value of the 100 pF was obtained at a rms voltage of 10 V with the classical calibration chain and only at 0.3 V with the DJIB calibration chain. No correction was applied for the voltage dependence which is estimated to be smaller than 0.006 *μ*F/F for this type of capacitor [7].

#### 2) Frequency Dependence of 1 nF:

**Figure 9.**
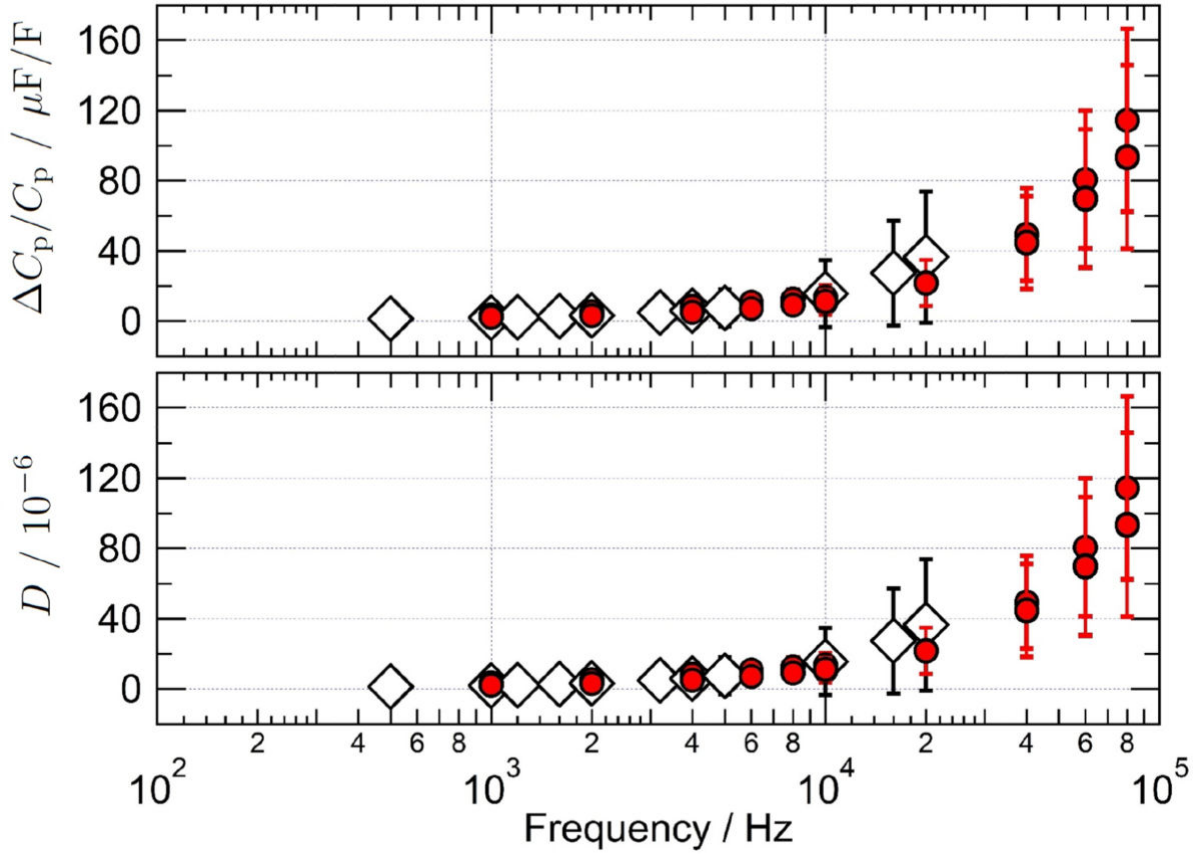
Calibration of 1 nF capacitance standards carried out at different frequencies, either with the DJIB (solid symbols) or using a commercial capacitance bridge, Andeen-Hagerling 2700A (open symbols). Both the relative deviation of the parallel capacitance from the nominal value (top plot) and the dissipation factor (bottom plot) were calibrated. The uncertainty bars correspond to the combined uncertainty (*k* = 1).

A 1 nF capacitance standard was calibrated over the frequency range from 1 kHz to 80 kHz by comparing it to various resistance standards. Depending on the frequency, three different resistance standards were used (1.2906 kΩ, 12.906 kΩ and 100 kΩ) to keep the impedance ratio |*Z*_1 nF_*/Z*_R_| between 0.1 and 10. The relative frequency dependence of the capacitance as well as the dissipation factor are shown in Fig. 9. The results of the DJIB measurements agree with the values obtained using a calibrated commercial capacitance bridge (Andeen-Hagerling AH2700A) up to its maximum measuring frequency of 20 kHz with much smaller measurement uncertainties. Moreover, the frequency range of the DJIB is a factor of four larger than that of the AH bridge.

The excellent agreement of the results obtained with the different calibration procedures confirms the ability of the DJIB to perform high accuracy R-C comparison over a broad frequency range.

### C. R-L comparison

As a final demonstration of the DJIB ability to compare any kind of impedance, inductance standards have been calibrated by direct comparison to resistance standards.

#### 1) Inductance calibration at 1 kHz:

**Figure 10.**
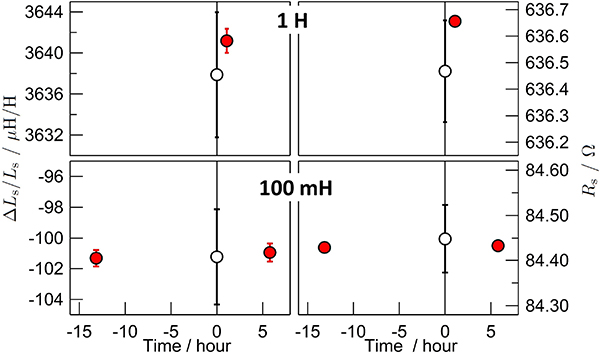
Calibration of 100 mH and 1 H inductance standards carried out at 1 kHz, either with the DJIB (solid symbols) or using the sampling system (open symbols) [55]. Both the relative deviation of the series inductance from the nominal value (on the left side) and the series resistance (on the right side) have been calibrated. The uncertainty bars correspond to the combined uncertainty (*k* = 1). The horizontal axis is the time delay, in hours, from the sampling measurement.

The calibration of 100 mH and 1 H inductance standards were performed at the frequency of 1 kHz with both the DJIB and the synchronous sampling system [55], routinely used at METAS for the realization of the inductance scale [6]. Both the series resistance and the deviation of the series inductance from the nominal value are shown in Fig. 10. To avoid stability and drift issues, all of the measurements were performed on the same day. However, the inductance standards had to be moved because the two measurement systems are not located in the same laboratory. The 100 mH standard was first measured with the DJIB, then measured with the sampling system and finally measured again with the DJIB. The 1 H standard was measured only once with each system.

These inductance standards are not temperature regulated. Therefore, an uncertainty related to the temperature stability, estimated to be about 0.1 K, has been included in the uncertainty of the sampling measurement. The two calibration procedures give the same results within the combined standard uncertainty (*k* = 1). The uncertainty obtained with the DJIB is approximately 5 times smaller than that obtained with the sampling system. However, this point has to be validated by future inter-laboratory comparison.

#### 2) Frequency dependence of 100 mH:

**Figure 11.**
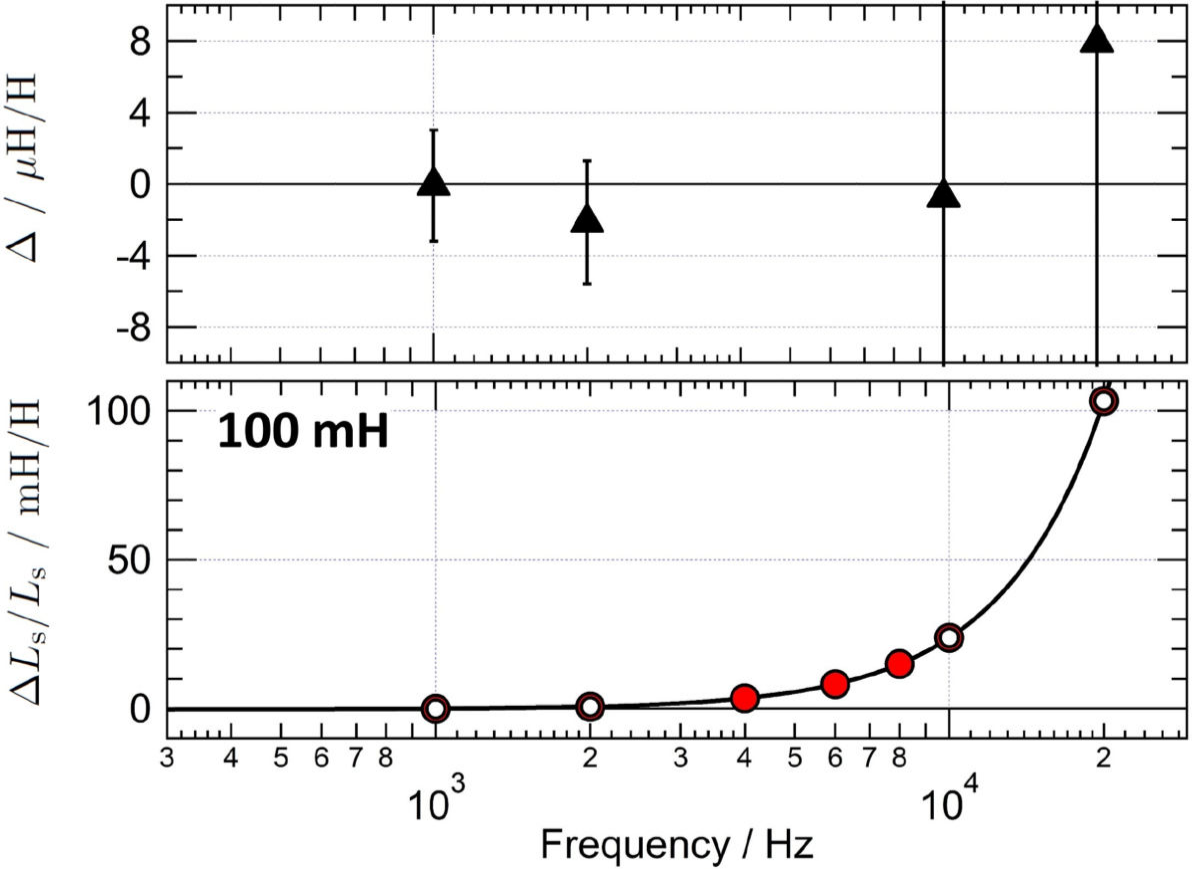
Bottom: frequency dependence of a 100 mH standard measured either with the DJIB (solid symbols) or with the sampling system (open symbols) [55]. The solid line is a quadratic fit of the DJIB measurements which is shown only as a guide for the eye. Top: the difference of between the DJIB and sampling measurements. The uncertainty bars correspond to the combined uncertainty of the sampling measurement alone (*k* = 1).

A further validation test of the DJIB has been carried out by measuring a 100 mH inductance standard at different frequencies between 1 kHz and 20 kHz. The bottom plot of Fig. 11 shows the frequency dependence of the relative difference of the inductance from its value at 1 kHz measured both with the DJIB (solid symbols) and with the sampling system (open symbols) [55]. The measured series inductance increases with frequency because of the internal interwinding capacitance that forms a resonant LC circuit [56]. The difference between the results of the two measurement methods is given on the top plot. The uncertainty bars correspond to the combined uncertainty of the sampling measurement alone (*k* = 1). The good agreement between the two sets of measurements clearly demonstrates the ability of the DJIB to perform such R-L calibration over a broader frequency range.

## VI. Conclusion

The Dual Josephson Impedance Bridge (DJIB) performs high accuracy comparisons of impedances defined as four terminal-pair standards at frequencies between 1 kHz and 80 kHz. The first prototype of the DJIB showed the potential of this type of bridge [18]. In this paper, a new upgraded DJIB has been developed and fully characterized. The different validation tests described in this paper clearly demonstrate the broad capabilities of the DJIB in high accuracy comparisons of impedance standards of any kind. In particular:

- Resistance comparisons were successfully performed at different ratios (1:1, 1:10 and 1:1.29). In addition, the high accuracy of the measurements performed above 10 kHz have raised critical questions regarding the calculability of the frequency dependence of the Haddad resistors fabricated at METAS.
- The calibration of a 100 pF capacitance standard performed at 1233 Hz with the DJIB is in good agreement with the result obtained with the classical calibration chain. The use of the DJIB makes realizing the R-C chain simpler (only one bridge is required) and faster (less than 2 hours of measurement instead of more than 7 hours) without compromising the measurement accuracy.
- The ability of the DJIB to calibrate inductance standards in terms of resistance has also been validated. The results are in good agreement with those obtained with the sampling system presently used for realizing the inductance scale.

In addition to its principal function of comparing impedance standards, the DJIB is also a sensitive tool for investigating the behavior of the JAWS sources. Indeed, quantum locking range measurements can easily be performed with high precision, uncovering potential sources of error in the output voltage delivered by the JAWS sources.

In a near future, we expect that improvements to the DJIB will further improve impedance metrology at frequencies above 10 kHz and will contribute to a better evaluation of the accuracy of the JAWS sources at those frequencies.

## Figures and Tables

**Table I:** Uncertainty budget of the DJIB for different frequencies. The uncertainties are given as relatives uncertainties in *μ*Ω/Ω with a coverage factor of *k* = 1

1 to 1 impedance ratio			

Uncertainty component	1 kHz	10 kHz	80 kHz
(a) Bridge resolution	0.009	0.013	0.028
(b) Cable correction	0.004	0.006	0.222
(c) Voltage injection chain	<0.001	<0.001	0.032
(d) Quantum locking range	<0.001	<0.001	<0.001

Combined	0.010	0.015	0.226

1 to 10 impedance ratio			

Uncertainty component	1 kHz	10 kHz	80 kHz

(a) Bridge resolution	0.024	0.024	0.055
(b) Cable correction	0.004	0.006	0.222
(c) Voltage injection chain	<0.001	<0.001	0.032
(d) Quantum locking range	0.024	0.075	0.266

Combined	0.035	0.079	0.354

**Table II:** List of calculable resistance standards used for resistor comparison measurements.

Name	Type	Nominal Value

G1	Quadirfilar	12.906 kΩ
G2	Quadrifilar	12.906 kΩ

H1a	Haddad	1.2906 kΩ
H1b	Haddad	1.2906 kΩ

H2a	Haddad	1 kΩ
H2b	Haddad	1 kΩ

**Figure 12.**
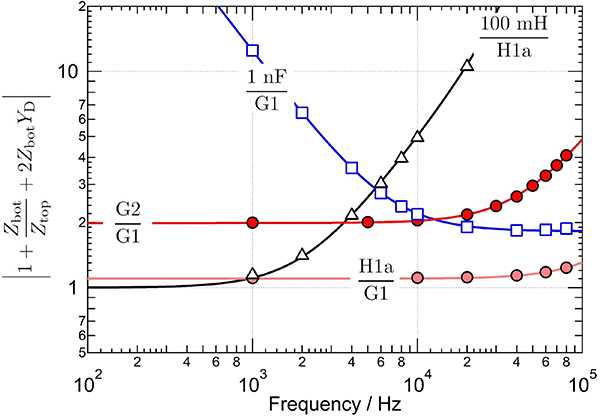
Modulus of the sensitivity coefficient of the voltage ratio on the main balance *D*_MB_ for different impedance comparison: R-R comparison in a 1:1 (G2 / G1) and 1:10 (H1a / G1) ratios. R-C comparison (1 nF / G1) and R-L comparison (100 mH / H1a). The symbols correspond to the measured values and the solid lines correspond to the model given by Eq. B.8. The only fitting parameter of the model is the stray capacitance *C*_D_ of the admittance *Y*_D_. The fitting parameter *C*_D_ changes from 280 pF to 385 pF depending on the impedance standards compared.

**Figure 13.**
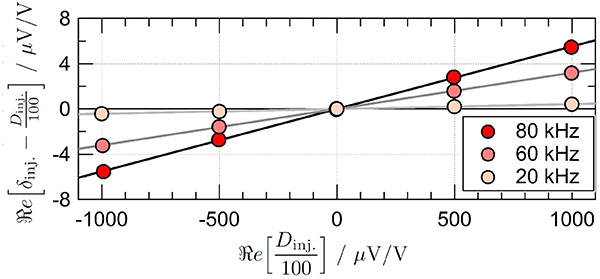
Measurement of the real part of the injection chain error at 20 kHz, 60 kHz and 80 kHz.

**Figure 14.**
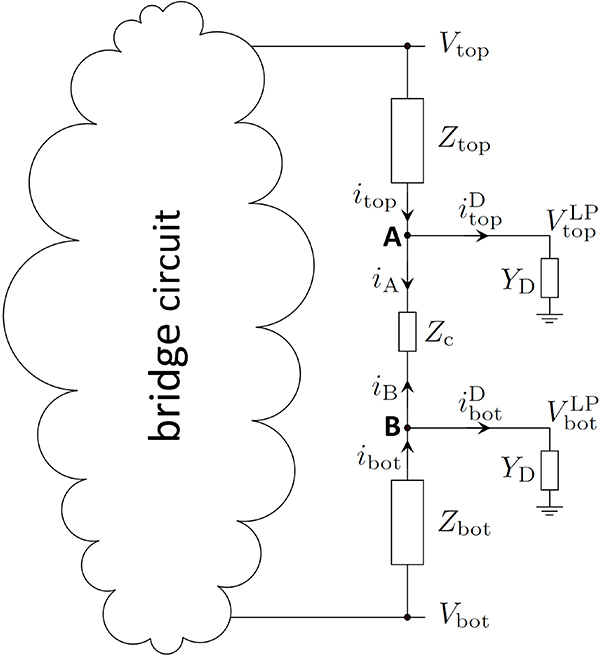
Equivalent circuit of a four terminal-pair bridge.

## Footnotes

1 Commercial instruments are identified in order to adequately specify the experimental procedure. Such identification does not imply recommendation or endorsement by NIST, nor does it imply that the equipment identified is necessarily the best available for the purpose.

## Appendix A Uncertainty components

A full evaluation of the different uncertainty components of the bridge has been performed in the frequency range from 1 kHz to 80 kHz. In this section, only the major uncertainty components dominating the uncertainty budget are reported.

### A. Bridge Resolution

The bridge resolution determines the smallest variation of the impedance ratio (i.e. of the voltage ratio) that can be observed. This parameter depends firstly on the noise level of the different balances (*D*_MB_, *D*_K_, *D*_top_ and *D*_bot_) and secondly on the effect of each of these balances on the determined voltage ratio (see Appendix B–A for notation).

The noise level of the DJIB is very similar to the noise level previously observed on the DAB [10]. When comparing two resistance standards of 12.906 kΩ, the main balance shows a white noise characteristic and the level of noise after 100 seconds of measurement is about 2 nV/V to 3 nV/V. This noise level is barely dependent on the frequency and is in fact related to the Johnson noise of resistance standards. When comparing resistance standards of smaller value, the noise level decreases to about 1 nV/V. The Kelvin balance also shows a white noise characteristic and the noise level is well below 1 nV/V after 100 seconds. The two auxiliary balances *D*_top_ and *D*_bot_ show a similar noise which is limited by the 1/*f* noise characteristic of the sources *S*_top_ and *S*_bot_ [55]. The output noise level of the sources is typically of order 10 *μ*V/V. However, using an auxiliary impedance *Z* of about 10 kΩ in the current arms of the bridge (see Fig. 1) greatly reduces the effect of the source’s output noise on the auxiliary balances [18]. Therefore, the noise level measured by the detector *D*_top_ and *D*_bot_ is typically of order 0.1 *μ*V/V at 1 kHz and increases to about 1 *μ*V/V at 80 kHz. If the resistance of the auxiliary impedance *Z* is reduce to the 100 Ω, the noise on the auxiliary balances increases to 5 *μ*V/V at 1 kHz.

The effect on the voltage ratio of a residual bias (or noise) in the main balance *D*_MB_ can be either calculated from the elements of the measured sensitivity matrix **M** or determined from the equivalent circuit of the bridge.

Fig. 12 shows the multiplying factor, $\left|1+\frac{Z_{\text{bot}}}{Z_{\text{top}}}+2Z_{\text{bot}}Y_{\text{D}}\right|$ needed to evaluate the effect of the main balance’s bias for different impedance comparisons (R-R, R-C and R-L). The good agreement between the measured values (symbols) and calculated values (lines) confirm the validity of the equivalent circuit (see Fig. 14) and the state equation Eq. B.8.

### B. Cable Correction

There are two types of cable corrections to take into account. The first is the loading effect of the cable between the on-chip voltage outputs of the JAWS sources and the detection transformers on the HP arms, where *V*_top_ and *V*_bot_ are defined. The second is the classical correction related to the cables needed to connect the impedance standards to the bridge [2], [20]. When the standards are exchanged at the level of the connectors of the standards, the connecting cables are part of the bridge and the effects of both cable corrections are removed by combining the ‘direct’ and ‘reverse’ measurement results (see Eq. B.13). Nevertheless, an uncertainty has to be evaluated to account for possible variation of cable characteristics between the two phases of the measurement. This uncertainty includes differences in the standards’ internal cables (section 5.4.4 in [2]), though in this paper that effect is small because the internal cables are short and equal.

For each channel of the dual JAWS there are two coaxial cables between the JAWS chip’s outputs and the head of the probe. The inner of one coaxial cable is connected to the high output of the JAWS chip and the inner of the other cable is connected to the low output of the JAWS chip. The inner and outer of the coaxial cable connected to the low are shorted together at the probe head [57]. The high output coaxial cable is therefore supplying the Josephson voltage to the bridge through a third cable from the probe’s head to the detection transformer in the bridge. The exact electrical parameters of the whole cable are not precisely known, but only the stability of these parameters have to be evaluated. These cables are not touched or connected/disconnected during the two phases of the measurement procedure. Only a small variation in the characteristics of the cables inside the probe is expected because of small changes in the liquid helium level in the dewar between the ‘direct’ and the ‘reverse’ measurements. In particular, the resistance of the cable is expected to change with liquid helium level and thus temperature. The capacitance and the inductance are expected to be barely dependent on the liquid helium level. Therefore, we conservatively estimate a stability of 0.1 Ω for the resistance, 3 nH for the inductance and 0.2 pF for the stray capacitance.

The evaluation of the cable correction related to the four terminal-pair definition of the standards requires the determination of the characteristics of the high potential (HP) cable (between the HP port of the standard and the detection transformer) and the LC cable (between the LC port of the standard and the injection transformer of the Kelvin source *S*_K_). Again, an accurate determination of the cable parameters is not required. Only the stability of these parameters has to be evaluated. The main source of variation of the cable characteristic is related to disconnection/connection process when the position of the two standards *Z*_top_ and *Z*_bot_ are reversed. Based on previous experience with similar cables and connectors, we conservatively estimate a stability of 0.1 mΩ for the resistance, 3 nH for the inductance and 0.5 pF for the capacitance.

### C. Voltage Injection Chain

The main balance of the bridge is obtained by adjusting the amplitude and the phase of *V*_bot_ by either modifying the Josephson voltage $V_{\text{ch2}\;}^{\text{JAWS}\;}$ or injecting a small voltage component *δ*_inj._. Neglecting all other corrections, the impedance ratio can be written as:

(A 1) $$\frac{Z_{\text{bot}\;}}{Z_{\text{top}\;}}=-\frac{V_{\text{ch}2}^{JAWS}}{V_{\text{ch}1}^{JAWS}\left(1-\delta_{\text{inj}}\right)}.$$

One of the dominant uncertainty component of the DAB [10] at high frequency is related to the fact that the actual injected voltage component *δ*_inj._ could differ from the measured quantity *D*_inj._*/*100. This error could be due either to a gain error and phase shift of the 100:1 injection transformer or to any deviation of the measured *V*_REF_ from *V*_bot_. The classical calibration of the whole injection chain is possible but it is time consuming process that requires the use of calibrated capacitance and resistance standards [2].

Using the unique ability of the DJIB to generate arbitrary voltage ratios, the calibration of the whole injection chain is straightforward. Indeed, the bridge can be balanced repeatedly using different Josephson voltages $V_{\text{ch2}\;}^{\text{JAWS}\;}$ that require the injection of different voltage components *δ*_inj._. Fig. 13 shows the real part of the injection chain error $ℜe\left[\delta_{\text{inj}.\;}-D_{\text{inj}.\;}/100\right]$ for different frequencies. The gain error of the injection chain is about 6 · 10^−3^ at 80 kHz.

To limit the uncertainty component related to the injection chain, the Joesphson voltage, $V_{\text{ch2}\;}^{\text{JAWS}\;}$, is adjusted during the procedure to guarantee that the main balance is obtained with a small injected voltage |*D*_inj._*/*100| < 10 *μ*V/V.

## Appendix B Detailed Description

### A. Balancing procedure and data processing

Each AO channel *x* generates a single tone voltage, *S*_*x*_, at the angular frequency *ω* = 2*πf*:

(B.1) $$S_{x}=\left[a_{x}\cos(\omega t)+b_{x}\sin(\omega t)\right]\cdot \sqrt{\left(2\right)}\cdot V_{\text{Nom}}^{\text{rms}}$$

where $V_{\text{Nom}}^{\text{rms}}$ is the rms value of the nominal voltage applied to the bottom impedance *Z*_bot_. The phase and amplitude of the generated voltage can be independently adjusted by modifying the dimensionless parameters *a*_*x*_ and *b*_*x*_. The same formalism is also applied to the output voltages of the dual JAWS source.

Due to the properties of the Fourier transform (FT), the FT of the voltage generated by the source *S*_*x*_ leads to

(B.2) $$\frac{\text{FT}{\left[S_{x}\right]}_{f}}{V_{\text{Nom}}^{\text{rms}}}=a_{x}-jb_{x}$$

where $j=\sqrt{-1}$. It is worth pointing out the negative sign before the imaginary part of the FT. This sign will have to be taken into account when calculating the initial settings of the sources *S*_top_ and *S*_bot_ as well as in the determination of the JAWS voltage ratio which is therefore given by

(B.3) $$\frac{V_{\text{ch}2}^{JAWS}}{V_{\text{ch}1}^{JAWS}}=\frac{a_{\text{ch}2}-jb_{\text{ch}2}}{a_{\text{ch}1}-jb_{\text{ch}1}}$$

where *a*_ch1_, *b*_ch1_, *a*_ch2_ and *b*_ch2_ are the calculated parameters of the quantum-based JAWS voltages.

There are 6 AI channels measuring the voltages at the different nodes of the bridge. Each digitizer simultaneously samples *N*_ADC_ values of the voltage with a sampling frequency *f*_ADC_. The duration of one data set is therefore given by *N*_ADC_*/f*_ADC_ and contains *P* = *N*_ADC_ · *f/f*_ADC_ periods of the measured signal. The amplitude and phase of the fundamental component of each measured signal is then calculated from the FT of the data sets. To avoid spectral leakages and to guarantee the accuracy of the FT calculation, *N*_ADC_ and *f*_ADC_ are chosen so that *P* is an integer (coherent sampling). Moreover, the sampling frequency must satisfy the Nyquist criterion *f*_ADC_ ≥ 2 · *f*.

For each digitizer, the quantity of interest *D* is finally calculated by normalizing the measured voltage by the reference voltage *V*_REF_. For example, the voltage measured by the digitizer *V*_inj_ leads to the measured quantity *D*_inj_:

(B.4) $$\frac{\text{FT}{\left[V_{\text{inj}}\right]}_{f}}{\text{FT}{\left[V_{\text{REF}}\right]}_{f}}=A_{\text{inj}}+jB_{\text{inj}}$$

Similarly, the voltages measured by the remaining four digitizers give the following measured quantities: $D_{\text{top}\;}^{\text{HP}}\equiv D_{\text{top}\;}$, $D_{\text{bot}}^{\text{HP}}\equiv D_{\text{bot}}$, $D_{\text{top}\;}^{\text{LP}}$ and $D_{\text{bot}\;}^{\text{LP}}$. In addition, two supplementary quantities are also defined: $D_{\text{MB}}\equiv \frac{1}{2}\left[D_{\text{top}\;}^{\text{LP}}+D_{\text{bot}}^{\text{LP}}\right]$ which is usually called the main balance and $D_{\text{K}}\equiv \frac{1}{2}\left[D_{\text{top}\;}^{\text{LP}}-D_{\text{bot}}^{\text{LP}}\right]$ usually called the Kelvin balance.

The balancing procedure is now reduced to the following problem: adjusting the source vector **S** = [*a*_top_ *b*top *a*_bot_ *b*_bot_ *a*_inj_ *b*_inj_ *a*_K_ *b*_K_]^*T*^ in such a way that all components of the measured vector **D** = [*A*_top_ *B*_top_ *A*_bot_ *B*_bot_ *A*_MB_ *B*_MB_ *A*_K_ *B*_K_]^*T*^ are zero. Using the quasi-Newton method [58], the balance is obtained in an iterative process where the new values **S**_*i*+1_ are calculated from the previous vectors **S**_*i*_ and **D**_*i*_ using the following relation:

(B.5) $$S_{i+1}=S_{i}-M_{\text{E}}^{-1}\cdot D_{i}$$

where **M**_E_ = **M**◦**E** is the Hadamard product (or element-wise product) of the sensitivity matrix defined as:

$$M=\left(\begin{array}{cccc} \partial A_{\text{top}\;}/\partial a_{\text{top}\;} & \partial A_{\text{top}\;}/\partial b_{\text{top}\;} & \cdots & \partial A_{\text{top}\;}/\partial b_{\text{K}}\\ \partial B_{\text{top}\;}/\partial a_{\text{top}\;} & \partial B_{\text{top}\;}/\partial b_{\text{top}\;} & \cdots & \partial B_{\text{top}\;}/\partial b_{\text{K}}\\ \vdots & \vdots & \ddots & \vdots \\ \partial B_{\text{K}}/\partial a_{\text{top}} & \partial B_{\text{K}}/\partial b_{\text{top}\;} & \cdots & \partial B_{\text{K}}/\partial b_{\text{K}} \end{array}\right)$$

and the selecting matrix **E** defined as:

$$E=\left(\begin{array}{llllllll} 1 & 1 & 0 & 0 & 0 & 0 & 0 & 0\\ 1 & 1 & 0 & 0 & 0 & 0 & 0 & 0\\ 0 & 0 & 1 & 1 & 0 & 0 & 0 & 0\\ 0 & 0 & 1 & 1 & 0 & 0 & 0 & 0\\ 0 & 0 & 0 & 0 & 1 & 1 & 0 & 0\\ 0 & 0 & 0 & 0 & 1 & 1 & 0 & 0\\ 0 & 0 & 0 & 0 & 0 & 0 & 1 & 1\\ 0 & 0 & 0 & 0 & 0 & 0 & 1 & 1 \end{array}\right)$$

The convergence of the bridge balance is faster when the matrix **M**_E_ is used instead of the sensitivity matrix **M**, like in the digitally assisted bridge (DAB) [10]. This convergence optimization is especially efficient when unlike impedance standards are compared at frequencies higher than 20 kHz. This automated balancing procedure is in fact very similar to a manual procedure where one source is adjusted to balance one detector, as represented by the Table III.

**Table III:** List of adjustment required to balance the bridge

generator		balance condition

*S*_bot_	⇒	*D*_bot_ = 0
*S*_top_	⇒	*D*_top_ = 0
*S*_inj_	⇒	*D*_MB_ = 0
*S*_K_	⇒	*D*_K_ = 0

### B. State Equation

While the impedance ratio is directly given by Eq. 1 when the bridge is perfectly balanced (i.e. the balance conditions of Table III are fulfilled), it is still useful to determine the state equation when the bridge is slightly out-of-balance.

Fig. 14 represents the equivalent circuit of the four terminal-pair bridge shown in Fig. 1. Applying the Kirchoff’s current law at the nodes **A** and **B** leads to

(B.6) $$\{\begin{array}{l} i_{\text{A}}=i_{\text{top}}-i_{\text{top}}^{\text{D}}=\frac{V_{\text{top}}-V_{\text{top}}^{\text{LP}}}{Z_{\text{top}}}-V_{\text{top}}^{\text{LP}}Y_{\text{D}}\\ i_{\text{B}}=i_{\text{bot}}-i_{\text{bot}}^{\text{D}}=\frac{V_{\text{bot}}-V_{\text{bot}}^{\text{LP}}}{Z_{\text{bot}}}-V_{\text{bot}}^{\text{LP}}Y_{\text{D}}. \end{array}$$

Summing the currents at the node, *i*_A_ + *i*_B_ = 0 and therefore

(B.7) $$\frac{Z_{\text{bot}\;}}{Z_{\text{top}\;}}=-\frac{V_{\text{bot}\;}}{V_{\text{top}\;}}[1-\frac{Z_{\text{bot}\;}}{Z_{\text{top}\;}}\frac{V_{\text{top}\;}^{\text{LP}}}{V_{\text{bot}\;}}-\frac{V_{\text{bot}\;}^{\text{LP}}}{V_{\text{bot}\;}}\;\;-Y_{\text{D}}Z_{\text{bot}}\frac{V_{\text{top}}^{\text{LP}}+V_{\text{bot}}^{\text{LP}}}{V_{\text{bot}}}]$$

which becomes

(B.8) $$\frac{Z_{\text{bot}\;}}{Z_{\text{top}\;}}=-V_{\text{ratio}\;}[1-\left(1+\frac{Z_{\text{bot}\;}}{Z_{\text{top}\;}}+2Z_{\text{bot}\;}Y_{D}\right)\cdot D_{\text{MB}}\;\;+\left(1-\frac{Z_{\text{bot}\;}}{Z_{\text{top}\;}}\right)\cdot D_{\text{KB}}]$$

when replacing *V*_bot_ by *V*_REF_ in the terms inside the brackets of Eq. B.7.

Equation B.8 is the state equation of the bridge which is reduced to Eq. 1 when *D*_MB_ = *D*_KB_ = 0.

### C. ‘Direct’ and ‘Reverse’ Measurements

As shown in Fig. 1, the voltages involved in the state equation Eq. 1 are the voltages defined at the center of the detection transformers along the HP arms of the bridge. Due to the cable loading effect [59]–[61], these voltages *V*_top_ and *V*_bot_ differ from the quantum-based reference voltages $V_{\text{ch1}\;}^{\text{JAWS}\;}$ and $V_{\text{ch2}\;}^{\text{JAWS}\;}$ generated at the output of the JAWS chips. Indeed, it can be shown [18] that, when the bridge is balanced:

(B.9) $$V_{\text{top}\;}=\frac{V_{\text{ch}1}^{\text{JAWS}}}{1+Y_{\text{c}}\left[Z_{\text{o}}+Z_{\text{c}}/2\right]}=V_{\text{ch}1}^{\text{JAWS}}\left(1+\Delta_{\text{ch}1}^{\text{load}}\right)$$

where *Z*_o_ is the output impedance of the JAWS chip and *Z*_c_ and *Y*_c_ are respectively the series impedance and the stray admittance of the cable. $\Delta_{\text{ch}1}^{\text{load}}$ is a complex number that describes the loading correction to apply to the calculated reference voltage $V_{\text{ch1}\;}^{\text{JAWS}\;}$ to obtain the defined voltage *V*_top_. The real part of the loading correction increases with the square of the frequency and the square of the cable length. The magnitude of the correction can reach a few 100 *μ*V/V at 80 kHz [62].

A similar correction applies to the bottom voltage and the impedance ratio is therefore given by

(B.10) $$\frac{Z_{\text{bot}\;}}{Z_{\text{top}\;}}=-\frac{V_{\text{ch}2}^{\text{JAWS}}\left(1+\Delta_{\text{ch}2}^{\text{load}\;}\right)}{V_{\text{ch}1}^{\text{JAWS}}\left(1+\Delta_{\text{ch}1}^{\text{load}\;}\right)}=-V_{\text{ratio}}^{\text{d}}\left(1+\Delta_{\text{load}\;}\right)\;\;=-\frac{a_{\text{ch}2}^{\text{d}}-jb_{\text{ch}2}^{\text{d}}}{a_{\text{ch}1}^{\text{d}}-jb_{\text{ch}1}^{\text{d}}}\left(1+\Delta_{\text{load}\;}\right)$$

where $a_{\text{ch}1}^{\text{d}}$, $b_{\text{ch}1}^{\text{d}}$, $a_{\text{ch}2}^{\text{d}}$ and $b_{\text{ch}2}^{\text{d}}$ are the calculated parameters of the quantum-based JAWS voltages and $\Delta_{\text{load}\;}\approx \Delta_{\text{ch}\;2}^{\text{load}\;}-\Delta_{\text{ch}1}^{\text{load}\;}$ is a systematic bias of the calculated voltage ratio due to the cable loading effect.

The determination of Δ_load_ would require the precise measurement of the cable specifications (*Z*_c_ and *Y*_c_) and JAWS chip output impedance *Z*_o_ of each JAWS voltage source. However, due to the unique ability of the dual JAWS sources to generate accurate voltages with arbitrary amplitude and phase, the bridge ratio measurement can be repeated with the position of the two impedance standards in the bridge reversed. The new state equation is then

(B.11) $$\frac{Z_{\text{top}\;}}{Z_{\text{bot}\;}}=-\frac{V_{\text{ch2}\;}^{\text{JAWS}\;}\left(1+\Delta_{\text{ch2}}^{\text{load}\;}\right)}{V_{\text{ch1}\;}^{\text{JAWS}\;}\left(1+\Delta_{\text{ch1}\;}^{\text{load}\;}\right)}=-V_{\text{ratio}\;}^{\text{r}}\left(1+\Delta_{\text{load}}\right)\;\;=-\frac{a_{\text{ch2}\;}^{\text{r}}-jb_{\text{ch2}\;}^{\text{r}}}{a_{\text{ch}1}^{\text{r}}-jb_{\text{ch}1}^{\text{r}}}\left(1+\Delta_{\text{load}}\right)$$

These two state equations can finally be combined to determine the cable loading effect

(B.12) $$1+\Delta_{\text{load}\;}=\frac{1}{\sqrt{V_{\text{ratio}\;}^{\text{d}}\cdot V_{\text{ratio}\;}^{\text{r}}}}$$

and the effective impedance ratio

(B.13) $$\frac{Z_{\text{bot}\;}}{Z_{\text{top}\;}}=-\frac{V_{\text{ratio}\;}^{\text{d}}}{\sqrt{V_{\text{ratio}\;}^{\text{d}}\cdot V_{\text{ratio}\;}^{\text{r}}}}=-\frac{V_{\text{bot}}}{V_{\text{top}\;}}=-V_{\text{ratio}}.$$

The ability of the DJIB to perform ‘direct’ and ‘reverse’ measurements for any impedance ratio greatly simplifies the characterization of the bridge. Indeed, many systematic biases cancel out after combining the two balances [63] as shown in Eq. B.13. The only requirement on the systematic biases is that they must be the same in both measurements i.e. the systematic bias scaling factor of each channel has to be independent of the amplitude and phase of the generated JAWS voltages. If the voltage ratio bias Δ_d_ in the ‘direct’ measurement is not exactly the same as the voltage ratio bias Δ_r_ in the ‘reverse’ measurement (for example, if the internal cabling of the impedance standards is significantly different), then a supplementary systematic bias term in Eq. B.13 will have to be taken into account and the effective impedance ratio will then be given by

(B.14) $$\frac{Z_{\text{bot}\;}}{Z_{\text{top}\;}}=-\frac{V_{\text{ratio}\;}^{\text{d}}}{\sqrt{V_{\text{ratio}\;}^{\text{d}}\cdot V_{\text{ratio}\;}^{\text{r}}}}\sqrt{\left(1+\Delta \right)}$$

where Δ ≈ Δ_d_ − Δ_r_

### D. Pulse Amplitude Slope: ‘Direct’ and ‘Reverse’ Measurements

Let us say that there is some offset error voltage to the realized JAWS voltage because of the JAWS bias pulses:

(B.15) $$V_{\text{top}\;}=V_{\text{ch}1}^{\text{JAWS}}+V_{\text{ch}1}^{\text{pulses}\;}$$

If we assume that at the synthesis frequency (1) there is an indirect, complex, linear coupling *α* between the bias signal *V*_bias_ and the voltage output leads, (2) that this bias signal is linearly dependent on the voltage being generated by the JAWS array $V_{\text{bias}\;}=\beta V_{\text{ch}}^{\text{JAWS}}$ with a (complex) slope *β*, and (3) that the bias signal is also linearly dependent on the pulse amplitude where the first two assumptions are taken at the optimum/default pulse amplitude and also valid for changes *δ* in the pulse amplitude, then the offset error voltage is given by

(B.16) $$V_{\text{ch}}^{\text{pulses}}=\alpha V_{\text{bias}}=\alpha \beta \cdot V_{\text{ch}}^{\text{JAWS}}(1+\delta )$$

and combining the initial constants into a slope *αβ* = *s*_ch_ (and re-labeling for the different channels)

(B.17) $$V_{\text{ch}}^{\text{pulses}}=s_{\text{ch}}\cdot V_{\text{ch}}^{\text{JAWS}}\left(1+\delta_{\text{ch}}\right)$$

and so going back to the equation for *V*_top_, under the above assumptions and definitions:

(B.18) $$V_{\text{top}\;}=V_{\text{ch}1}^{\text{JAWS}}\left[1+s_{\text{ch}1}\left(1+\delta_{\text{ch}1}\right)\right]$$

Combining this equation with a similar equation for *V*_bot_ and channel 2, the ratio in the ”direct” configuration is now

(B.19) $$\frac{Z_{\text{bot}\;}}{Z_{\text{top}\;}}=-\frac{V_{\text{ch}2}^{\text{JAWS}\;}\left[1+s_{\text{ch}2}\left(1+\delta_{\text{ch}2}\right)\right]}{V_{\text{ch}1}^{\text{JAWS}}\left[1+s_{\text{ch}1}\left(1+\delta_{\text{ch}1}\right)\right]}$$

where the ratio of errors can be simplified under the further assumption that *s*_ch_ ≪ 1 using:

(B.20) $$\Delta_{\text{dither}\;}=-1+\frac{\left[1+s_{\text{ch}\;2}\left(1+\delta_{\text{ch}\;2}\right)\right]}{\left[1+s_{\text{ch}\;1}\left(1+\delta_{\text{ch1}\;}\right)\right]}\;\;\approx s_{\text{ch}2}\left(1+\delta_{\text{ch}2}\right)-s_{\text{ch}1}\left(1+\delta_{\text{ch}1}\right)$$

and so

(B.21) $$Z_{\text{ratio}\;}=\frac{Z_{\text{bot}\;}}{Z_{\text{top}\;}}=-\frac{V_{\text{ch}\;2}^{\text{JAWS}\;}}{V_{\text{ch1}\;}^{\text{JAWS}\;}}\left(1+\Delta_{\text{dither}}\right)\;\;=-V_{\text{ratio}\;}^{\text{d}}\left(1+\Delta_{\text{dither}}\right)$$

where typical ratio variables have also been introduced.

Similarly in the ‘reverse’ configuration,

(B.22) $$\frac{1}{Z_{\text{ratio}\;}}=\frac{Z_{\text{top}\;}}{Z_{\text{bot}\;}}=-\frac{V_{\text{ch}2}^{\prime }\text{JAWS}}{V_{\text{ch}1}^{\prime \text{JAWS}}}\left(1+\Delta_{\text{dither}}\right)\;\;=-V_{\text{ratio}\;}^{\text{r}}\left(1+\Delta_{\text{dither}}\right)$$

assuming that Δ_dither_ has not changed which is equivalent to assuming that the same pulse amplitude dithers *δ*_ch1_ and *δ*_ch2_ have been used during the ‘direct’ and ‘reverse’ measurements and that the coupling constants *s*_ch1_ and *s*_ch2_ have not changed (it is fine and expected for either/both pattern magnitudes $V_{\text{ch}\;}^{\text{JAWS}}$ to change).

Then collecting the *Z*_ratio_ for the two configurations:

(B.23) $$Z_{\text{ratio}\;}=-\frac{1}{V_{\text{ratio}\;}^{\text{r}}\left(1+\Delta_{\text{dither}}\right)}\;\;=-V_{\text{ratio}\;}^{\text{d}}\left(1+\Delta_{\text{dither}}\right)$$

And calculating the combined ratio starting with the two equations for the ‘direct’ *Z*_ratio_ and ‘reverse’ 1*/Z*_ratio_,

(B.24) $$1=V_{\text{ratio}\;}^{\text{d}}V_{\text{ratio}\;}^{\text{r}}{\left(1+\Delta_{\text{dither}}\right)}^{2}\;1+\Delta_{\text{dither}\;}=\frac{1}{\sqrt{V_{\text{ratio}\;}^{\text{d}}\cdot V_{\text{ratio}\;}^{\text{r}}}}\;Z_{\text{ratio}\;}=-\frac{V_{\text{ratio}\;}^{\text{d}}}{\sqrt{V_{\text{ratio}\;}^{\text{d}}\cdot V_{\text{ratio}\;}^{\text{r}}}}$$

which shows that this type of error voltage due to the feedthrough of the JAWS bias pulses will not effect the measured ratio when using the reversal procedure and under the initial assumptions (1)-(3).

However, if Δ_dither_ changes between the ‘reverse’ and ‘direct’ configurations, then

(B.25) $$Z_{\text{ratio}\;}=-\frac{V_{\text{ratio}\;}^{\text{d}}}{\sqrt{V_{\text{ratio}\;}^{\text{d}}\cdot V_{\text{ratio}\;}^{\text{r}}}}\sqrt{\frac{(1+\Delta_{\text{dither}}^{\text{d}}}{(1+\Delta_{\text{dither}}^{\text{r}}})}\;Z_{\text{ratio}\;}\approx -\frac{V_{\text{ratio}\;}^{\text{d}}}{\sqrt{V_{\text{ratio}\;}^{\text{d}}\cdot V_{\text{ratio}}^{\text{r}}}}\left(1+\frac{\Delta_{\text{dither}\;}^{\text{d}}}{2}-\frac{\Delta_{\text{dither}\;}^{\text{r}}}{2}\right)$$

and this type of JAWS error voltage can cause an error in the measured ratio even when using the reversal procedure.
